# The Therapeutics of the Respiratory Passages

**Published:** 1885-05

**Authors:** 


THE THERAPEUTICS OF THE RESPIRATORY PASSAGES. By Prof. James, M. D. New York: William Wood & Co., 56 and 58 Lafayette Place. 1884.

This work claims consideration as a whole, and appeals to the medical practitioner for a thorough perusal of its contents; but it is clear that many will be deterred from such a task by the array of that Hippocratic lore in the outset which indicates some want of a correct appreciation of the practical element.

A further examination of the valuable and elaborate disquisitions upon the various measures of management for the derangements of the vitalizing function of respiration, must impress the reader with the great research and acumen of the author.

We are therefore inclined to modify the unfavorable indications, so far as to warrant a scrutiny of the separate chapters by those who may not be able to take them up *seriatim*, as much profit may be derived from a study of such points as may be of special interest to the physician who needs information upon one or another topic included in this volume.

